# Co-Creating Descriptors and a Definition for Person-Centred Coordinated Health Care: An Action Research Study

**DOI:** 10.5334/ijic.5575

**Published:** 2021-03-03

**Authors:** Amanda Phelan, Daniela Rohde, Mary Casey, Gerard Fealy, Patrick Felle, Gabrielle O’Kelly, Helen Lloyd, Aine Carroll

**Affiliations:** 1 Trinity College, Dublin, Ireland; 2 University College Dublin, Ireland; 3 University of Plymouth, UK

**Keywords:** person centred co-ordinated care, integrated care, patient narratives, action research

## Abstract

The aim of this study was to co-create a definition and generic descriptors for person-centred coordinated care for Ireland generated from service users’ narratives. An overarching action research approach was used to engage and empower people to tangibly impact health policy and practice. Through focus groups and a qualitative survey, primary data were collected from a national sample of health services users, caregivers and health care service users’ representative groups. Thematic analysis was used to analyse the data. Three major themes were co-produced as essential care elements. These were: ‘My experience of healthcare’, ‘Care that I am confident in’ and ‘My journey through healthcare’. Through an IPPOSI partner project steering group and their membership groups’ contribution, these themes were further refined into a definition of person-centred coordinated care and nineteen related generic descriptors. Key findings demonstrate that within complex, fragmented healthcare systems, the subjective expectations of service users should be integrated into care delivery, with a scaffolding of services to meet service users’ needs between care settings and disciplines and over time.

## Introduction

In recent years, healthcare providers, policy makers and regulators have focused on consumer engagement and empowerment in order to improve standards of care delivery, provide effective, efficient care services and promote quality improvement [1]. This emphasises that healthcare needs to be safe, person-centred, timely and equitable [2], meeting specific targets set out in the United Nations Sustainable Development Goals [3]. Current care challenges include compartmentalized, uncoordinated care [4], leading to service gaps and poor consumer confidence [5]. What is required is a partnership model of care, wherein quality care is scaffolded around the individual underpinned by organisational change [6,7]. This reorientation centres on ‘real-world’ planning, which integrates structural inequalities of care through meaningful co-design approaches [8,9]. The empowerment of consumers of healthcare in structuring and delivering integrated services is essential for quality, safe and cohesive health systems [10]. Moreover, service users’^1^ experience and satisfaction are increasingly considered valuable indicators of person-centred care [6,11].

In 2017, the Irish Health Service Executive (HSE) embarked on a new programme of reform with the development of new integrated care programmes. A founding tenet of the programmes was taking the user perspective as the main organising principle and so, through the Patient Narrative Project,^2^ the HSE commissioned a study to elicit the essential elements of people’s expectations of the health service. Built on the principles of public and service user engagement, the study was conducted in partnership with caregivers and service user representatives with the aim of co-designing and co-creating a definition of person-centred coordinated care (PCCC) and developing a set of descriptors in the form thematic domains and constituent ‘I’ statements.

## Irish Healthcare System and Policy Directions

In the past, the Irish healthcare system has often been described as a Beveridge-type system. However, funding and provision is, in reality, a mixed system. Although approximately three in every five people have private healthcare insurance, this only contributes 15% to the overall health budget. Overall responsibility for the health and social care system lies with the Government, exercised through the Department of Health, under the direction of the Minister of Health with the formation of the HSE in 2005 with delegation to provide public health and social care nationally.

In 2017, the *Sláintecare* Report [12] proposed a radical ten-year plan of healthcare reform leading to universal healthcare, targeting four work streams: service redesign and supporting infrastructure; safe care, coordinated governance and value for money; teams of the future; and sharing progress. Although *Sláintecare* offered a roadmap to a universal, single-tier person centred health service, the *Sláintecare Strategy and Action Plan* [13] did not fully commit to a legislative underpinning [14].

Globally, there has been a greater focus on stakeholder engagement in policy development [15,16] and in Ireland, public consultation and engagement are seen as important when developing and implementing health policy. Eliciting feedback on the experience of care is considered a fundamental part of service quality improvement with global initiatives such as those by the Picker Institute [17]. In Ireland, the results of the national patient experience survey, which yielded over 12,000 responses from forty hospitals, indicated overall high scores in service users’ satisfaction. However, despite the quality improvement focus of *Sláintecare*, almost 40% of respondents reported that they did not always have enough time for discussion with their doctor and 55% indicated that they were not informed about medications’ side effects while long waiting lists for treatment persisted [18]. Thus, the *Patient Narrative Project’s* [19] fundamental aim was to build trust and confidence in care by listening to service users’ experiences. This study reports on phase one of the four stage project focusing on the development of a definition and description of person-centred co-ordinated care.

## Person Centred Co-ordinated Care (PCCC)

Person centredness is an increasing focus in contemporary healthcare. For example, there has been a transformation towards humanizing care, where the perspectives, values and beliefs of the person are central. Harding et al. [20] proposes person centred care as an overarching group of concepts (shared decision-making, integrated care, self-management), an emphasis on individual personhood and partnership with other attributes being identified as enabling flourishing, leadership, collaborative approaches and empowerment [21]. Person or relationship centred models (***Table 1*** provides examples) have guided care delivery based on shared decision making and partnership.

**Table 1:** Frameworks/Models of Person or Relationship Centred Cultures.


FRAMEWORK/MODEL	DOMAINS/COMPONENTS

*Senses’ Framework [22]*	Centres around the concept of relationship centred care and includes families, older people and staff. There are six domains: 1) Sense of security, 2) Sense of belonging, 3) Sense of continuity, 4) Sense of purpose, 5) Sense of achievement and 6) Sense of significance.

*Person Centred Practice Framework [21]*	Framework with the person at the centre of care. Five domains: 1) Macro-context, 2) Pre-requisites, 3) Care environment, 4) Person-centred processes and 5) Person Centred outcomes.

*Gothenburg Model [23]*	Three components: 1) Partnership, 2) Patient narrative/story and 3) Documentation

*Person centred care conceptual framework [24]*	Based on Donabedian’s model [25] Structure-Healthcare systems/Organisational level Process: Healthcare Provider levels Outcome: Patient-healthcare provider-healthcare system/organisational level.


Although the term concept of care ‘centredness’ emphasizes care built around the person, there are discrete differences. While the term person centred care has been used interchangeably with patient centred care, Eklund et al., [26], suggest person centred care’s goal is to foster a meaningful life for the patient, while patient centred care is concerned with maximising functional capacity. Person centredness has also emerged in relation to particular populations such as older people [22], people living with dementia [27], general practice [28] and healthcare [21]. McCormack et al. [29] points to person centredness being a culture of care defined as:

“… an approach to practice established through the formation and fostering of healthful relationships between all care providers, service users and others significant to them in their lives. It is underpinned by values of respect for persons, individual right to self-determination, mutual respect and understanding. It is enabled by cultures of empowerment that foster continuous approaches to practice development”. (29:3)

PCCC represents an integrated care system where the individual’s choice, values, will and preference are core to care delivery, with the integration of social capital within the context of community-centred approaches [30]. Building on theories of person-centred cultures [21,31], PCCC recognises that people rarely experience health care as a singular entity. Rather, the care journey is temporal and characterised by interfacing different settings, different professionals and support systems via horizontal and vertical integration.

The complexity of contemporary health systems demands a revision of existing fragmented care provision [6,9,32], recognising the wider social determinants of health [33]. The aim of PCCC is to improve professional contact, treatment and follow-up through effective care coordination and integration. This can result in the democratization of health, enhanced collaboration, shared leadership, reduced hospital admissions and effective discharge planning [32]. PCCC underpins the focus of Ireland’s healthcare reform agenda, with its focus on achieving a single-tier health system [12].

Despite the stated advantages of PCCC, there can be difficulties in defining and measuring the concept and its impact on patient outcomes [1]. Measures to record experiences of PCCC in routine practice and work on developing a PCCC Organisational Change Tool [30] have been reported. Additional evidence is also needed on the effectiveness of PCCC, in terms of reducing demand on healthcare resources [32]. However, focusing on immediate cost benefits or overt health outcomes may obscure longer-term, less tangible benefits, such as user experience and reported outcomes, such as subjective wellbeing and quality of life [34]. To make PCCC a reality, researchers, commissioners, service providers and service users need to work together to create timely and sustainable change [1].

## The Study

The aim of the study was to engage with service users, caregivers and service user representatives to co-create and co-develop a definition (and generic descriptors) of PCCC in Ireland. Ethical approval was obtained from the Human Research Ethics Committee at University College Dublin. Thus, similar to work in the United Kingdom [35], the participants became the ‘active educators’ [36] in what is necessary for person centred co-ordinated care.

## Research Methods

**Figure 1:**
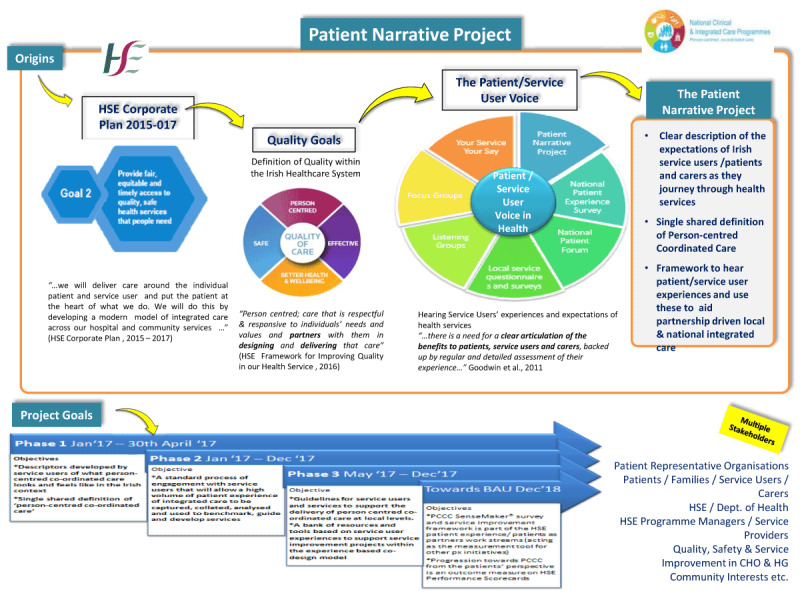
The phases of the patient narrative project.

This paper reports on Phase 1 of a project to develop PCCC as shown in ***Figure 1***.

Working in partnership with an umbrella patient led organisation (Irish Platform for Patient Organisations, Science and Industry (IPPOSI)) and the HSE, the study applied a participatory action research (PAR) approach using mixed methods. IPPOSI has a membership of over 100 different groups that work to put patients at the heart of health innovation. We define PAR as an iterative approach to research or learning that actively involves the populations being researched as agents of change [37]. PAR happens within a cyclic pattern and originates from the fields of adult education, international development, and the social sciences [38]. It is recognized as a more inclusive form of inquiry and can be viewed as a way of “bringing participation into action research” [39: 127].

This PAR study applied co-design principles with dialogue between the researchers, patients and advocates through IPPOSI and the HSE throughout the process. PAR is underpinned by cycles of reflecting, planning and action through a relational, reflexive process of mutual engagement [40]. The overall aim for the project was active stakeholder involvement as co-researchers to co-create a shared definition of PCCC as well as a set of descriptors. The democratic involvement of ‘knowing subjects’ [41] empowers participants to frankly discuss perspectives, creating living knowledge within the practical realm.

**Figure 2:**
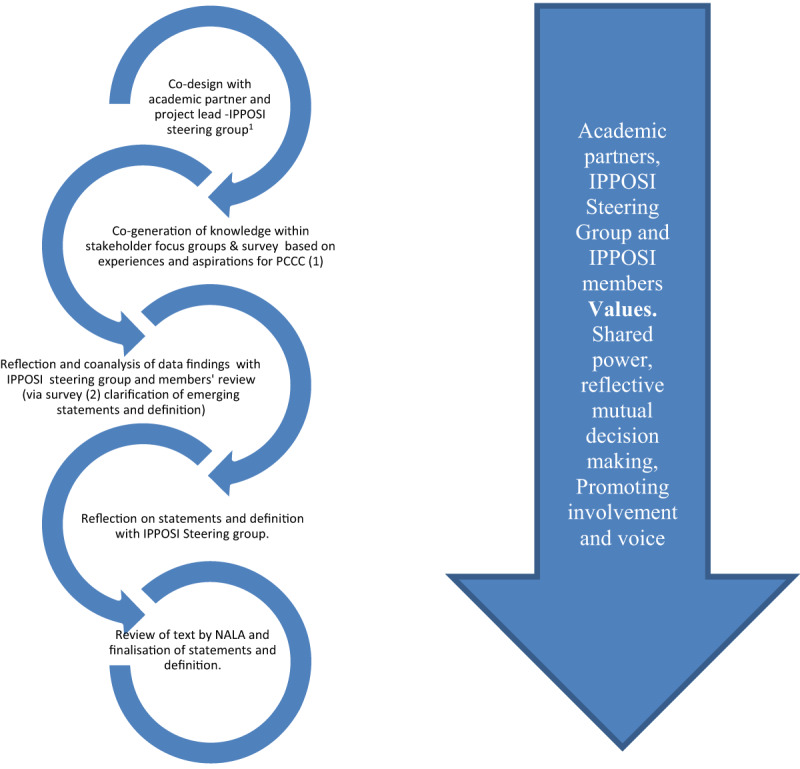
Iterative process of PAR in the Patient Narrative Project. ^1^ IPPOSI Steering Group included membership from the HSE.

From the study inception, public and healthcare service user involvement was key, whereby IPPOSI established the study aims and parameters, including the preferred methodology, through a call for tenders to academic institutions. The organisation also acted in both governance and facilitator roles, holding three meetings of the Patient Narrative Steering Group with the academic partner as well as, acting as a gatekeeper in supporting the recruitment of constituent service user and representative groups (see *https://www.hse.ie/eng/about/who/cspd/patient-narrative/progress-achievements/*). The iterative PAR process involved cycles of reflection, action and feedback, in the context of the democratic, active involvement of service users, caregivers and representative organisation in reviewing and refining what PCCC should be. This process is presented in ***Figure 2***.

## Data Collection

Eleven focus groups in four different geographical areas of Ireland were convened in the first quarter of 2017. These groups were in regions (Cork, Dublin, Galway, Cavan) which supported a national representation and occurred in local hotels with two held in a cardiac foundation venue. IPPOSI and the HSE disseminated information to recruit adult participants who were supported to attend by the provision of travel expenses. Data collection was undertaken by nurse academics, (four females, one male) and a male medical academic; all were experienced in qualitative research. A prepared topic guide was used to guide the focus group and discussions were digitally recorded. The aim was to draw on the experiences and perspectives of participants to develop themes that translated to descriptors-that is, statements which described service user expectations of using the health service in Ireland. In this study, it was the reflections of past experience of and future aspirations for care that the participants spoke of engaging with the health service. Focus group views were paramount and both facilitators and moderators emphasized their role as co-researchers rather than bringing any disciplinary lens on the discussion.

A purposive sample was comprised of 78 adults, who used any aspect of the health care service, people who provided care for healthcare service users and patient representative groups. Focus groups have been used in participatory research and are useful at gaining an in-depth view on social issues, personal experiences and perceptions [42]. The process followed Morgan’s [43] focus group approach, where an interactive discussion was enabled by a skilled facilitator and an assistant. As described by Burrows and Kendall [44], the facilitator created a relaxed atmosphere and guided discussion, ensuring inclusivity, while the assistant observed group dynamics and picked up on non-verbal communication. Although saturation was not a goal of the data collection, the reflections of focus group members demonstrated numerous common experiences.

Focus groups were supplemented by a qualitative 12-item e-survey with IPPOSI member groups. The focus groups topic guide and the survey questions were based on an adaptation of the CAHPS® Patient Narrative Elicitation Protocol [45].

## Data Analysis

The focus groups were transcribed verbatim and the data were managed using NVIVO 10©. Braun and Clarke’s [46] thematic approach was used to synthesize the data into themes. Thematic analysis is a flexible approach which enables the review of multiple perspectives and is useful to identify key features within data [47].

Using NVIVO 10, two researchers separately undertook the initial coding and analysis and developed preliminary themes, which were then subject to agreement. In partnership with the study authors, the IPPOSI study steering group, critically reflected and refined the PCCC descriptors and the definition of PCCC (see ***Figure 2***). When agreement was achieved, the statements and the definition were reviewed by the National Adult Literacy Agency to maximise understanding.

## Results

The analysis of narratives constituted three broad themes and sub-themes. Themes represented a ‘care without walls’ approach where care is integrated, person-centred and collaboratively scaffolded around the person and their life world.

### My Healthcare Experiences

The ‘My healthcare experience’ theme represented a major relational focus. This had a number of sub themes, a) communication that is understandable to me, b) communication that provides me with the required information I need, c) care that understands my life world including those who care for me, d) care that demonstrates positive regard for me and e) care that is based on authentic partnership and respects my choices.

#### Communication that is understandable to me

Participants emphasized that communication from healthcare professionals needed to be presented in a way that respected cultural realities and delivered in an understandable way. For example, the traveller population, a distinct ethnic group in Ireland, spoke of healthcare professionals’ assumptions about literacy skills:

‘Well in the Traveller Movement, we have all that information but it’s to Travellers we give it out because Travellers [don’t] have a lot of literacy skills. And I would have been the first one of my family to have been educated.’ [FG6]

Other examples included experiences of the use of professional jargon or the lack of accommodation for people who had hearing or sight problems:

‘…they didn’t look for… an interpreter [for a deaf person], the doctor didn’t ask for one, [Person] was moved over to A and E, they didn’t look for one and now he’s on the ward and it is only now that that person has texted us.’ [FG 11]

#### Communication that provides me with the required information I need

Information on practical issues related to assistance in getting the supports which were fundamental to daily life:

‘Like domiciliary care allowance, nobody gets told about that or say, occupational therapy adaptations to cars, nobody knows about the VRT [Vehicle Registration Tax].’ [FG 1]

#### Care that understands my life world including those who care for me

Participants also detailed the complex worlds they inhabited, which had commitments, yet this was unappreciated, particularly in relation the inflexibility of hospital appointment times:

‘I have to say, I have the same problems where I ask the receptionist who gives the appointments, I say, ‘I have a sick child at home, I am sick too, can I have an appointment in the morning’ and it is just ‘Computer says no.’ [FG 1]

Even when attendance for an appointment was successfully negotiated, prolonged waiting proved challenging:

‘Our experience is of long waiting times for appointments, then long waiting times at appointments almost without exception…The wait can be 3 hours for a 10–15 minute appointment.’ [Survey]

#### Care that demonstrates positive regard for me

Within the healthcare service, one of the most valued areas articulated by the participants was being treated with dignity and respect. One participant remarked on the incredulity of having to remind staff of fundamental basic communication norms:

‘… [Hospital] did a poster, I think with the [Hospital] and it was ‘we’d like to introduce ourselves or we’d like to know who you are’. Please ask us to introduce ourselves’. Like the whole thing is you shouldn’t have to ask a doctor to introduce themselves.’ [FG 4]

This demeaning of the person could also be related to the healthcare professional not attending to what the person is saying, conveying a lack of positive regard:

‘I was starting to get cognitive problems and I really wasn’t listened to at all and it was me that had kind of find the way through the community and I had to find where I needed to go to.’ [FG 2]

#### Care that is based on authentic partnership and respects my choices

Following on from challenges in communication, participants also described how the person could have their voice marginalized and the demonstration of healthcare paternalism:

‘That’s [paternalism] probably in a lot of care actually, the doctor a lot of the time assumes they know best, which really isn’t always the case, they’re medical professional but they don’t always know best what’s personally for you.’ [FG 11]

So rather than being influenced and directed to a choice, authentic self-determinism was desired:

‘It is like, you know, I want to know what is on the shelf in the supermarket. What can I have, how much does it cost and how long will it take to get it?’ (FG 1)

### Healthcare I am Confident In

The experiences of the participants pointed to the need to have healthcare that inspired their confidence. This involved four sub-themes, namely a) staff that are competent in delivering my care, b) care that delivers me high quality and safe care, c) care that is accountable, and d) care where I experience continuity.

#### Staff that are competent in delivering my care

The participants expressed a need for professionals’ competencies for particular types of care provision:

‘At times, inexperience of professionals may be an issue, as is their familiarity with certain conditions.’ [Survey]

One participant detailed a difficult encounter of having her child being referred to a devolved hospital:

‘…So, if I wasn’t a health professional myself and able to speak up here…I would be driving up at midnight to an adult imaginary hospital for my child.’ [FG 1]

#### Care that delivers me high quality and safe care

Closely linked to competency was the need for staff to recognise their scope of practice and to refer on, if additional expertise was required:

‘That they [doctors] actually own up and say, ‘I actually don’t know’ and step aside and let you get to the guy who does know.’ [FG 1]

Quality and safe care could be impacted by service constraint and rationing. This could lead to methods of resisting:

‘Don’t take them [family member] out [of hospital] or you’re back to square one.’ [FG 7]

#### Care that is accountable

When the participants reflected on care, they pointed to the need to have accountable, responsible professionals:

‘Lack of accountability for decision-making by managers that impacts on daily life of person with disability.’ [Survey]

It was also noted that accountability was a positive focus as it led to service improvement:

‘It was because he was being held accountable and he knew it… that things got done now.’ [FG 4]

#### Care where I experience continuity

Continuity of care had two components. The first was the lack of continuity in staff, so visits were often followed up with different staff members. The second aspect was the need to then repeat the same information when meeting the new healthcare professional.

‘The interns keep moving and you might not get the same intern even though they are still here when you’re going to the clinic.’ [FG 10]

Having comprehensive and shared records that were actually read by staff was seen as fundamental to continuity of care for the person:

‘Get a proper system where all health care professionals have access to medical notes regarding the patient…So less time is wasted explaining EVERYTHING over and over again.’ [Survey]

### My Journey Through Healthcare

People rarely have a unidisciplinary or single setting healthcare experience. Three dimensions of care were articulated by the participants. These were a) care that has a holistic approach to my health and my world, b) co-ordination of my care in health and areas outside health and c) access to services when I need them.

#### Care that has a holistic approach to my health and my world

People live lives where they need to have healthcare scaffolded around them rather than healthcare being divorced from the context of their lives:

‘But she [older person] finally got a bed and when they had seen her they wanted to send her home. So, she [caregiver] said to them there was no way she could take her home because she wasn’t able to manage her. So finally anyway they kept her for another week…’ [FG 3]

#### Co-ordination of my care in health and areas outside health

When care was not co-ordinated and seen as an isolationist experience within disciplines and settings, care was experienced as disjointed.

‘I think sort of connection between primary care and secondary or tertiary care, connection I think you know connection in relation to information and communication, health professional or whatever you know.’ [FG 2]

It was suggested that a care champion should be available:

‘A nurse shouldn’t have to be liaising between patients and there should be a co-ordinator, I think, someone who’s educated on how to use the system and they take on a certain amount of people and they walk them through the system…’ [FG 4]

#### Access to services when I need them

A repeated issue for participants was the waiting lists to have treatment. This caused significant distress, disillusionment and deterioration of symptoms:

‘Our 7 year old son was diagnosed with scoliosis almost 18mths ago and no sign of an appointment. Also waiting on appointments for rheumatology, psychiatry and cardiology.’ [Survey]

There was a stark recognition that long waiting lists in the public system meant paying for private care could give a more comprehensive service:

‘Going privately seems to be more of a necessity to get services.’ [FG 3]

Also, availability could be geographically determined:

‘If we want respite [for MS] we have to go to [County] which is hard to get, they have only one bed or two beds.’ [FG 8]

All these factors contributed to a system which was under strain to provide care and more importantly, care when it was needed by individuals. This led to participants struggling with access to various healthcare professionals in all care areas, yet, access was expedited if the ability to pay for private care was possible.

**Figure 3:**
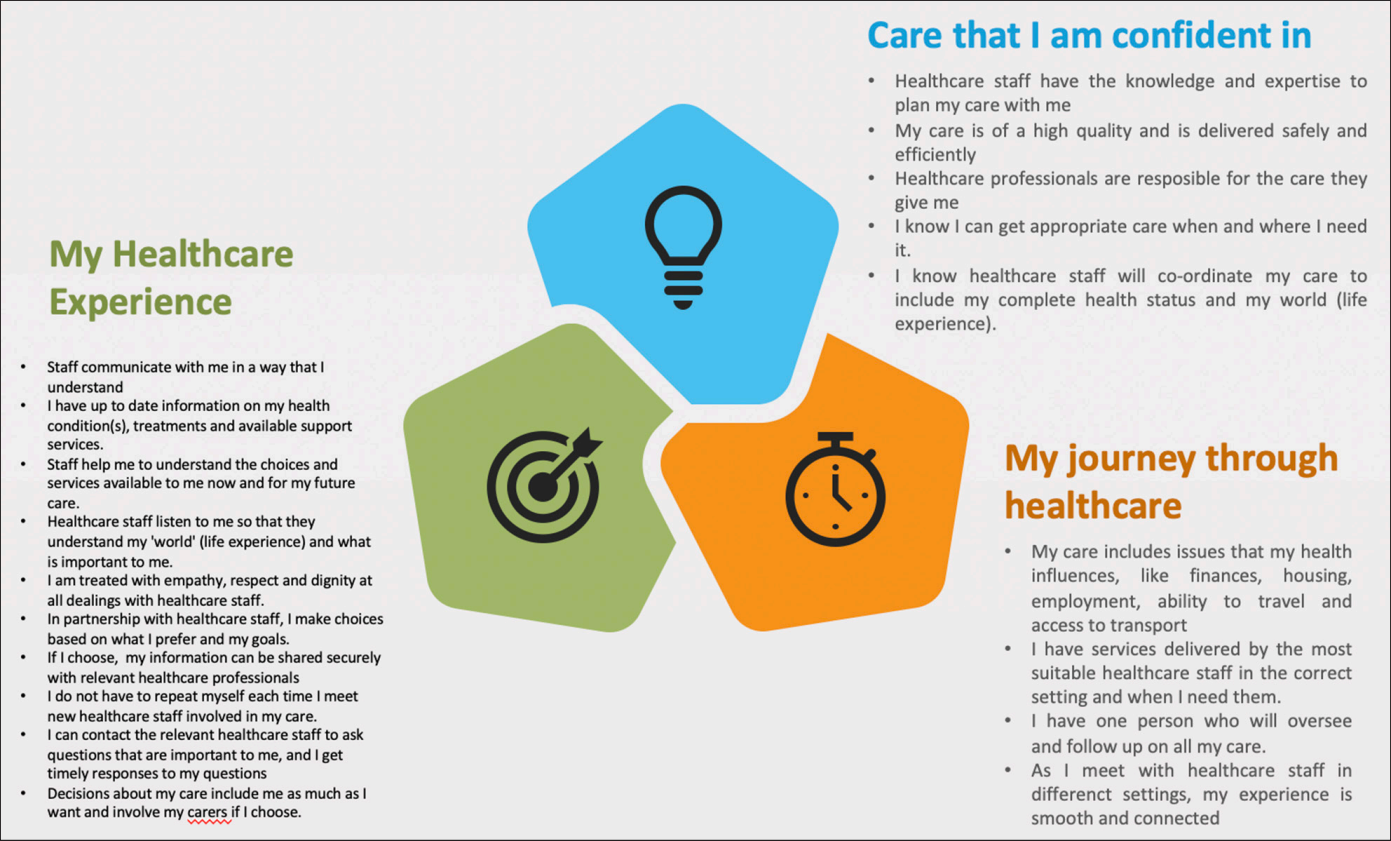
Statements describing PCCC.

The three themes were represented to the IPPOSI Steering groups with tentative ‘I’ statements and a definition of PCCC. These were also circulated to IPPOSI membership groups for review and feedback via an on-line survey. Following discussion, critical reflection and synthesis, 19 ‘I’ statements were agreed which represented PCCC in Ireland (***Figure 3***).

Concurrently with the development of the ‘I’ statements, the following definition of PCCC was refined through critical reflection with the IPPOSI steering group which drew upon a synthesis of the ‘I’ statements:

‘Person-centred coordinated care provides me with access to and continuity in the services I need when and where I need them. It is underpinned by a complete assessment of my life and my world combined with the information and support I need. It respects my choices, building care around me and those involved in my care.’

The definition reflected the essential aspects of integrated care which included being able to access services when needed, having a comprehensive care plan which enables care when and where it is needed. Most importantly, being person centred focused care on the individual and their perspectives rather than an inflexible system approach. Both the ‘I’ statements and definition were used in the second phase of the Patient Narrative Project to develop a standard process of engagement with service users through ‘Your Voice Matters’ survey (*https://www.hse.ie/eng/about/who/cspd/patient-narrative/your-voice-matters/_*).

## Discussion

This paper details phase one of the HSE patient narrative programme. In this study, the democratic nature of PAR enabled shared engagement and collaboration leading to the co-creation of experienced based descriptors on PCCC. Consequently, experiential knowledge is given equal status to empirical or practitioner knowledge [48]. Patient experience and patient satisfaction data is important to ensure organisational foci map to person-centredness not system centredness [49]. Such data can also demonstrate levels of care quality and service responsiveness [11]. The findings represented the foundation for further phases within the Patient Narrative Project (***Figure 1***).

There is a recognition of fragmentation in health service delivery and the need to meet public expectations of effectiveness, efficiency within person centred approaches [1]. Participatory action research is a feature many countries’ policy development [50] and involves the participation of those whose lives or work are impacted by the study focus [48]. Although there were positive accounts of experiencing healthcare, many participants detailed challenges. The way people were related to and communicated with was deemed particularly important and this represents an essential aspect of co-designed person centred care [21,31] yet remains challenging [18]. Communication needs to be dually effective; being enmeshed in the direct interaction with the person and between healthcare professionals. Essentially, the valuing of the person is demonstrated through a respect for their preferences, priorities and needs within the health system [35]. This represents a culture shift in healthcare from paternalism to person centredness [51]. Equally, only through the comprehensive provision of information can informed choice be articulated [5,52] and playing an active part in one’s own healthcare increases positive outcomes [53] while caregiver involvement is fundamental [35]. Thus, the scaffolding of care represents a delicate co-construction of care that matches the person’s values and beliefs and is flexibly built around him/her and the carers’ life world.

Confidence in care is also crucial in PCCC [11]. It is fundamental that care delivered is empowering, enhancing the value of service to the individual [54]. Competence is linked to person centred care, as well as inter-disciplinary collaboration, employing evidence-based practice, applying quality improvement mechanisms and integrating health informatics in care delivery [55]. Various serious case reviews have linked poor competency to sub-standard care outcomes and a disempowered patient population [16,56]. Consequently, as recognised by the participants, it is necessary to have clear systems of transparency and accountability [57].

Continuity of care was considered a key component of PCCC. Continuity of care is typified by a unified, consistent approach to meeting an individual’s health needs taking into account preferences and personal lifestyle [58]; it encompasses care quality [32] and provides security [58]. Informational continuity translates to the acquisition of information about past events [59], while management continuity refers to facilitating PCCC [58]. To avoid issues in accessing services and consequential stress [5], a focused collaboration is required and mutuality in care planning [11]. Equally, fostering relationships with the person receiving care is critical to partnership-based care experiences [9].

To achieve a health service that delivers what people want demands a reconfiguration of health services’ delivery [6]. Lessons can be learned in the process of developing such bespoke descriptors and definition. The context and structures of health, health policies and political agendas vary in every country, therefore applying a participatory approach has merit in generating an inductive response to quality in service users’ experience and engagement in healthcare. In Ireland, the need to meet public expectations and satisfaction has led to valuing the experiences of people. While a similar process occurred in the UK [35] and the definition and descriptors have common foci with this Irish study, there are also divergences, representing the mapping of population and person centred experiences, integrated at multiple levels [6]. This tallies with care planning efforts for structural reconfiguration which begun with six healthcare regions, single budgets based on need and an integration of care services [60]. However, ensuring policy is population focused, co-designed and context bound is central to meaningful implementation [9]. The *Slaintecare* [13] plan does offer promise in the context of a universal healthcare approach. Moreover, in a recent consultative process [10], submissions identified that integration of healthcare, a service organised around individuals’ needs, patient pathways and a more efficient use of services were key principles for service reform. In addition to a structural reconfiguration of services, the major focus of participants on relational aspects of care points to a deeper transformation in terms of culture and communication aspects of care. Financial support is also fundamental. Acknowledging the progress identified in the *Slaintecare* Action Plan [13], the OECD [14] note challenges regarding fiscal support for its implementation. This concern is undoubtedly exacerbated by the financial impact of the COVID-19 pandemic on the nation’s economy generally and the health budget specifically.

Limitations are acknowledged. The generalisations of the study may be limited to the Irish environment, however, there are parallels with similar international work. The study timeframe precluded the recruitment of ‘hard to reach’ populations, such as refugees or homeless people. In addition, we only recruited adults who had the capacity to consent and those who were empowered to speak. The experiences of children, those who are disempowered or people without functional capacity may also be different to our participants. Finally, the delivery of PCCC also has additional stakeholders such as healthcare professionals and policy makers, who may offer alternative insights regarding integrated care.

## Conclusion

Identification of the critical components of PCCC, the cost-effectiveness of providing or not providing these components and methods for implementation are essential if care providers are to recognise and rectify care delivery gaps [34]. This study provides a participatory world view of people’s experiences of healthcare services presented in a way that not only provides shared meaning but also a road map for change. As such, findings offer important preliminary insights for the development of PCCC health systems. While it is acknowledged that much work has been done, particularly in the context of health system reform within *Slaintecare* [13], health system reform needs to continue and be underpinned by measurements of service users’ experience for real world transformations. Additional research will enable an evaluation of the impact of PCCC related to service users’ involvement (co-design and co-development), experience, outcomes and continued service adaptability.

## Footnotes

Service user is a person who uses the healthcare services.

Narrative is used to describe the spoken account and not a narrative methodology.
